# Genome-wide characterization and comparison of endogenous retroviruses among 3 duck reference genomes

**DOI:** 10.1016/j.psj.2024.103543

**Published:** 2024-02-09

**Authors:** Yuan Bai, Yang Xi, Xinxin He, Grace Twumasi, Shengchao Ma, Qiuyu Tao, Mengru Xu, Shuaixue Jiang, Tao Zhang, Yinjuan Lu, Xu Han, Jingjing Qi, Liang Li, Lili Bai, Hehe Liu

**Affiliations:** ⁎ State Key Laboratory of Swine and Poultry Breeding Industry, College of Animal Science and Technology, Sichuan Agricultural University, P. R. Chengdu 613000, China; † Key Laboratory of Livestock and Poultry Multi-omics, Ministry of Agriculture and Rural Affairs, College of Animal Science and Technology, Sichuan Agricultural University, P. R. Chengdu 613000, China; ‡ Farm Animal Genetic Resources Exploration and Innovation Key Laboratory of Sichuan Province, Sichuan Agricultural University, P. R. Chengdu 613000, China

**Keywords:** duck, genome, gene, endogenous retrovirus

## Abstract

Endogenous retroviruses (**ERV**) are viral genomes integrated into the host genome and can be stably inherited. Although ERV sequences have been reported in some avian species’ genome, the duck endogenous retroviruses (**DERV**) genome has yet to be quantified. This study aimed to identify ERV sequences and characterize genes near ERVs in the duck genome by utilizing LTRhavest and LTRdigest tools to forecast the duck genome and analyze the distribution of ERV copies. The results revealed 1,607, 2,031, and 1,908 full-length ERV copies in the Pekin duck (ZJU1.0), Mallard (CAU_wild_1.0), and Shaoxing duck (CAU_laying_1.0) genomes, respectively, with average lengths of 7,046, 7,027, and 6,945 bp. ERVs are mainly distributed on the 1, 2, and sex chromosomes. Phylogenetic analysis demonstrated the presence of *Betaretrovirus* in 3 duck genomes, whereas *Alpharetrovirus* was exclusively identified in the Shaoxing duck genome. Through screening, 596, 315, and 343 genes adjacent to ERV were identified in 3 duck genomes, respectively, and their functions of ERV neighboring genes were predicted. Functional enrichment analysis of ERV-adjacent genes revealed enrichment for Focal adhesion, Calcium signaling pathway, and Adherens junction in 3 duck genomes. The overlapped genes were highly expressed in 8 tissues (brain, fat, heart, kidney, liver, lung, skin, and spleen) of 8-wk-old Mallard, revealing their important expression in different tissues. Our study provides a new perspective for understanding the quantity and function of DERVs, and may also provide important clues for regulating nearby genes and affecting the traits of organisms.

## INTRODUCTION

**Fig. 1:**
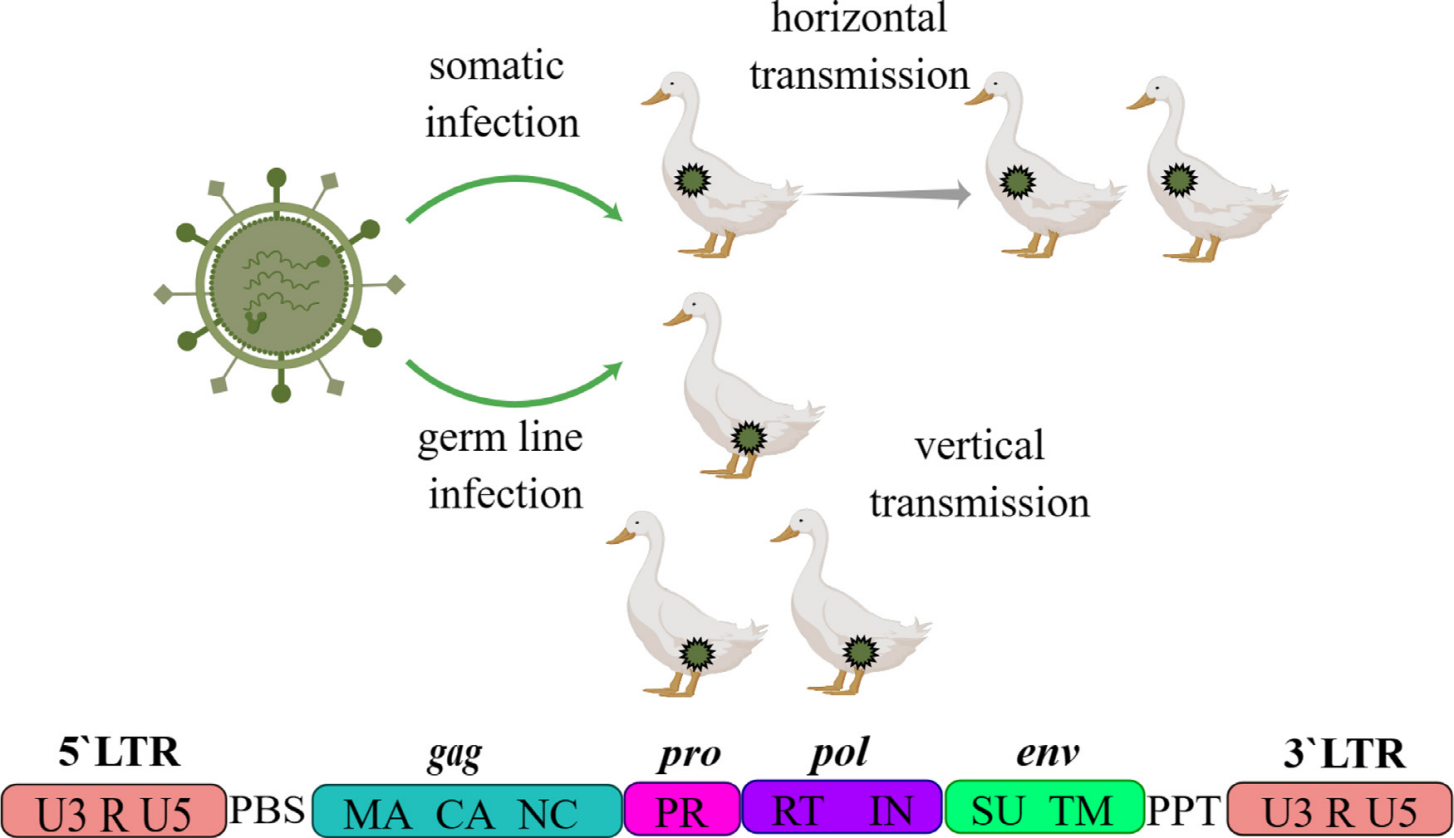
Structure of classical ERVs. Drawn by online software of Figdraw. (https://www.figdraw.com/static/index.html#/paint_index).

ERVs originate as retroviruses and have a unique genomic structure and mode of replication. When a retroviral RNA virus infects the cells of an organism, it usually inserts its genome into the host's genome in the form of reverse transcription to form a DNA form of the provirus, a process that, if it occurs and integrates into germ cells (Mourikis et al., 2016), has the potential to spread and become fixed in the genome of the entire species and can be passed on to the next generation. Such retroviruses embedded and integrated into the host genome are called ERVs and passed on to the next generation through the Mendelian pattern of inheritance (Bannert and Kurth, 2006). ERVs have a genome length of about 7000-12000 bp. Their genome structures are generally similar, with the typical structure being (Fig. 1): 5′LTR-pbs-gag-pro-pol-env-ppt-3′LTR (Menéndez-Arias et al., 2017), in which the 5′LTR and 3′LTR structures are the Long Terminal Repeat (**LTR**); the pbs structure is Primer Binding Site (PBS); the ppt structure is Polypurine Tract (PPT); and gag, pro, pol, env represent the key regulatory genes of retrovirus genome. The gag gene encodes the core proteins of retroviruses, such as capsid (**CA**), matrix (**MA**), and nucleocapsid (**NC**); the pro gene also encodes viral protease (**PR**); the pol gene encodes reverse transcriptase (**RT**) and integrase (**IN**); whereas the env gene encodes the surface membrane protein (**SU**) and transmembrane protein (**TM**) of retroviruses (Johnson, 2019).

Repetitive sequences derived from ERVs are widespread in the avian genome, ranging from 0.2 to 3.6%. However, the frequency of endogenous viral elements in the avian genome is still 6 to 13 times lower than in mammals (Cui et al., 2014). Most ERV insertions are incomplete fragments or long terminal repeats that occur alone and can regulate nearby genes (Jern and Coffin, 2008). Their insertion causes genes to undergo shear variation, epigenetic regulatory changes, replication and recombination (Galindo-González et al., 2017). In recent years, ERV insertion polymorphism mining and application research has gradually been emphasized in biodiversity, gene evolution, and breed identification research in livestock and poultry. Endogenous retroviruses have been extensively studied in poultry, but research on the duck genome has been limited. Among different poultry species, ducks are sturdy, prolific and disease resistant in nature (Patil et al., 2020). Therefore, this study aimed to compare the distribution and characterization of ERV in 3 different duck reference genomes using specific computational tools. The ERV sequence neighboring genes were searched so as to explore the potential function of ERV in the duck genome.

## MATERIALS AND METHODS

### ERVs Identification and Annotation

The study utilized genomic data from Pekin duck (ZJU1.0) GCF_015476345.1 downloaded from NCBI, while the China Agricultural University provided the genomic data and annotation files for Mallard (CAU_wild_1.0) and Shaoxing duck (CAU_laying_1.0) (Zhu et al., 2021). ERVs were identified in the genomes mentioned above using LTRhavest (Ellinghaus et al., 2008) and LTRdigest (Steinbiss et al., 2009) software integrated within GenomeTools. To search for retroviral genes in LTRdigest, we searched for Pfam libraries using the keywords “gag, protease, reverse transcriptase, ribonuclease H, integrase, env, dUTPase.” The Pfam library constructed by Steinbiss et al., 2009 was also referenced and supplemented, and a library containing 31 Pfam (Table S1) entries was finalized as an input to the LTRdigest for the detection of the structural domains of proteins encoded by the gag, pol, and env genes.

### Classification of ERVs

To improve the analysis of the acquired endogenous retroviral sequences and their classification into different genera, we chose 24 meticulously annotated viral sequences with full-length sequences as reference (Table 1). A phylogenetic tree was then constructed utilizing the GTR+G model with 1,000 confidence value tests, through comparison and maximum likelihood method using MEGA 7.0.14 (Kumar et al., 2016) software. In addition, we selected the top 30 ERVs with the most protein structural domains in the hits to construct a phylogenetic tree, eliminated ERVs at the level of some unplaced scaffolds, and finally identified 21, 24 and 24 ERVs (Table S2) in the genomes of Pekin duck (ZJU1.0), Mallard (CAU_wild_1.0) and Shaoxing duck (CAU_ laying_1.0), respectively. All ERV sequences were classified into distinct genera by comparing their similarity to known vertebrate ERV sequences.

**Table 1:** List of reference sequences used for phylogenetic analysis in this study.

classification	Retrovirus	GenBank accession	Length
*Alpharetrovirus*	*Rous sarcoma virus - Prague C*	J02342	9625
	*Rous sarcoma virus*	AF033808	9392
	*Fujinami sarcoma virus*	AF033810	4788
*Betaretrovirus*	*Mouse mammary tumor virus*	AF033807	8805
	*Jaagsiekte sheep retrovirus*	M80216	7462
	*Squirrel monkey retrovirus-H*	M23385	8785
*Deltaretrovirus*	*Human T-lymphotropic virus 2*	M10060	8952
	*Bovine leukemia virus*	AF033818	8419
	*Primate T-lymphotropic virus 1*	AF074966	9028
*Epsilonretrovirus*	*Walleye epidermal hyperplasia virus type 1*	AF014792	3603
	*Walleye epidermal hyperplasia virus type 2*	AF014793	3741
*Gammaretrovirus*	*Gibbon ape leukemia virus*	M26927	8088
	*Koala retrovirus*	AF151794	8431
	*Feline leukemia virus*	AF052723	8448
*Lentivirus*	*HIV-1*	AF033819	9181
	*Simian immunodeficiency virus*	M58410	9623
	*Bovine immunodeficiency-like virus*	M32690	8482
	*Feline immunodeficiency virus*	M25381	9474
	*Human immunodeficiency virus 2*	M30502	10359
	*Jembrana disease virus*	U21603	7732
*Spumaretrovirus*	*Simian foamy virus type 1*	X54482	12972
	*Equine foamy virus*	AF201902	12035
	*feline foamy virus*	AJ223851	10479
	*Human foamy virus*	Y07725	13242

### Acquiring Adjacent Genes to ERVs and Analyzing their KEGG and GO Functions

To enhance comprehension of the adjacent genes (There only needs to be an intersection between the start and end positions of both genes and ERVs) of ERV sequences, we initially utilized commands to pinpoint the positions of all genes within the duck genome via the Linux cluster. Subsequently, we extracted the intervals of ERV sequences and compared the positional data of the genes with that of the ERV sequences obtained from the duck genome. Finally, we retrieved the names of ERVs and genes with overlapping locations. Gene ontology (**GO**) and the Kyoto Encyclopedia of the Genome (**KEGG**) pathway enrichment analysis were performed at this site (http://www.bioinformatics.com.cn/), the screening criteria were *p-value* < 0.05 (Table S3).

### Gene Expression in Tissues

Expression profile of overlapped genes (Fig. 7G) analysis was performed using Heatmap tools in Hiplot Pro (https://hiplot.com.cn/), a comprehensive web service for biomedical data analysis and visualization. Gene expression data in tissues (brain, fat, heart, kidney, liver, lung, skin, and spleen) were provided by Duckbase (www.duckbase.org/expression/). These samples were sequenced on an Illumina HiSeq 2,500 system. Hisat2 (v2.1.0) (Li et al., 2019b) built the index of genome files and mapped RNA-seq data to the reference genome (duckbase.refseq.v4.fq). In the quality control step, clean reads were obtained by removing reads containing adapter, poly-N, and low-quality reads from raw data. Subsequently, the transcripts were assembled with Stringtie version 1.3.3b, and gene quantification was performed. After getting the raw readscount, use DESeq2 (https://www.bioinformatics.com.cn/) to normalize it to get the CPM value.

## RESULTS AND ANALYSIS

### Characterization of the Number, Distribution and Structure of ERVs in the Duck Genome

**Fig. 2:**
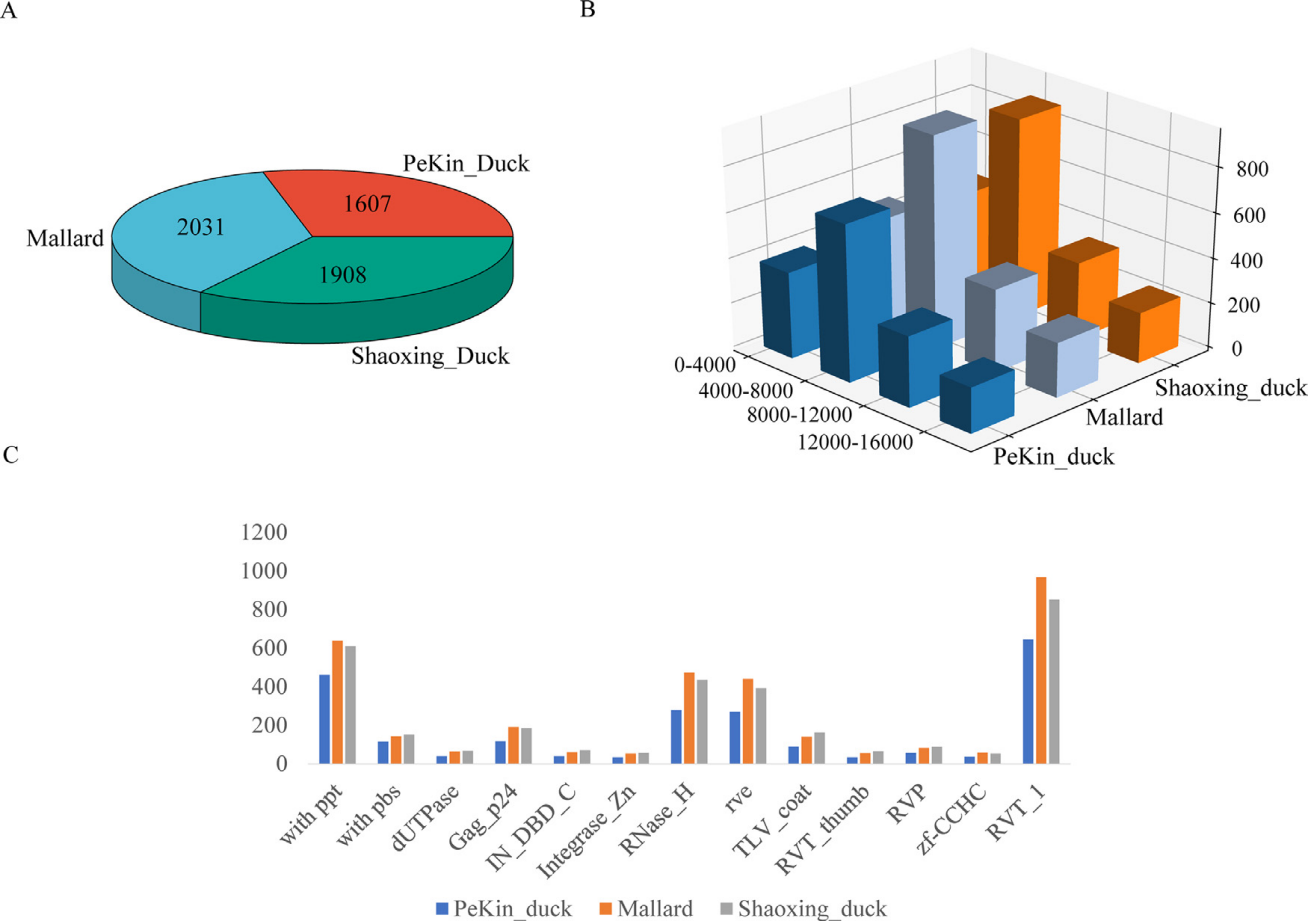
Number and structural characterization of ERVs in the duck genome. (A) Number of ERVs in the duck genome. (B) Distribution of ERV sequence lengths in the duck genome. (C) Comparison of ERV elements in the duck genome.

The structural features of ERVs involve long terminal repeats located at both ends of the ERV sequences, typically ranging between 100 and 1,000 bp in length. This feature plays a crucial role in identifying ERVs in the genome. A full-length ERV in the genome contains both a long terminal repeat sequence (**LTR**) and a target site repeat (**TSD**) at both ends. The region between the 2 long terminal repeats is known as the internal sequence of the ERV. We utilized LTRhavest to forecast endogenous retroviral sequences in the genomes of Pekin duck (ZJU1.0), Mallard (CAU_wild_1.0), and Shaoxing duck (CAU_laying_1.0). Filtering of ERVs not at the chromosome level, the results showed that 1607, 2031, and 1908 ERVs (Fig. 2A) were present in the 3 duck genomes (Table S4), respectively. The average length of endogenous retroviral sequences in the Pekin duck genome was 7,046 bp, while it was 7,027 bp in the Mallard genome, and 6,945 bp in the Shaoxing duck genome. From the results, the Mallard genome exhibited the highest count of ERVs, sequences with ERV lengths in the 4,000 to 8,000 bp range were the most common among the 3 duck genome versions (Fig. 2B). The number and the average length of endogenous retroviral sequences obtained in this study varied little among the 3 duck genomes.

Three characteristic sequences of ERVs in duck genomes were annotated using LTRdigest: primer binding sites (**PBS**), polypurine sequences (**PPT**), and protein structural domains. The results showed that endogenous retroviral elements such as PPT, PBS, RNase-H, rve and RVT-1 were more widely distributed in the 3 duck genomes. However, it should be noted that a high proportion of internal gene deletions were present in the endogenous retroviral sequences (Fig. 2C). With this approach, it appears that no intact functional ERVs were found among the detected DERVs. Most ERVs are no longer structurally intact due to accumulated mutations and recombination during hundreds of thousands of years of evolution.

**Fig. 3:**
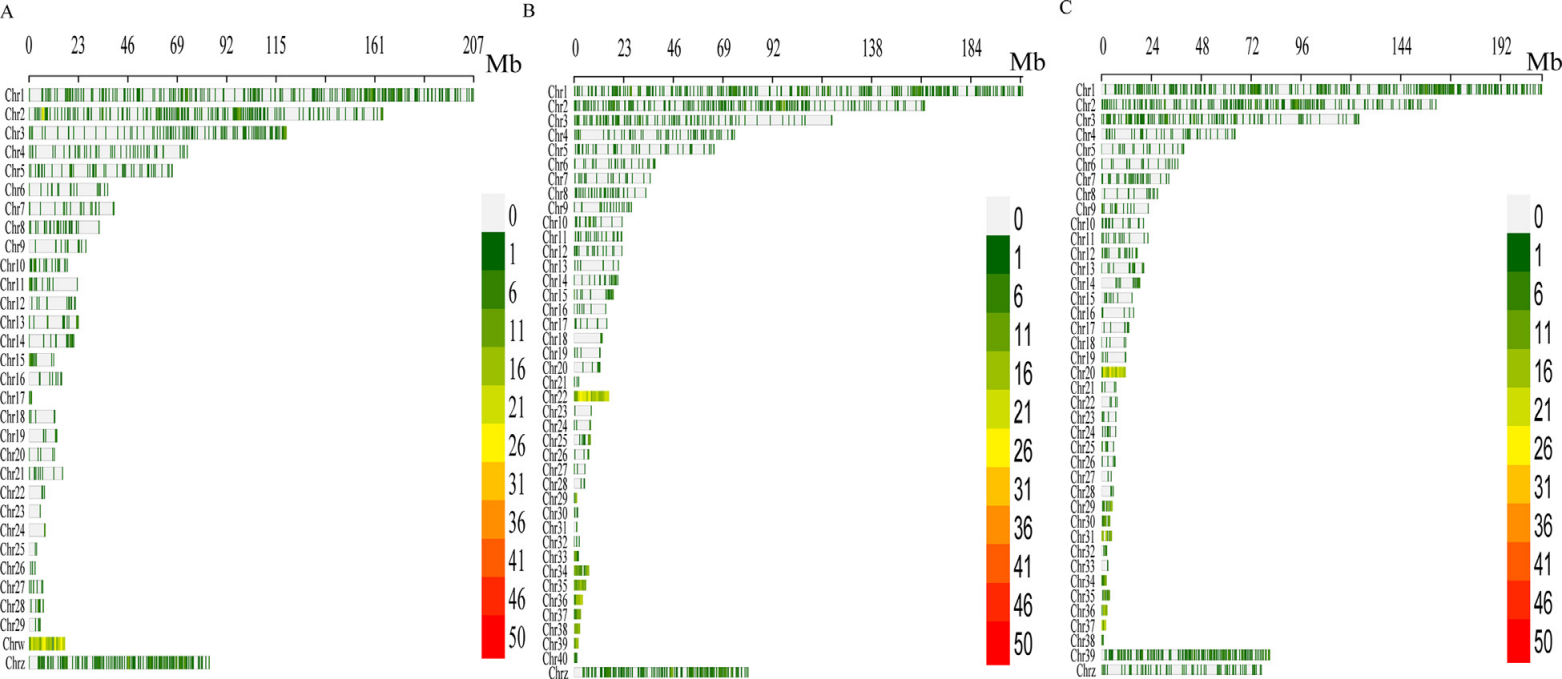
Distribution of ERVs in the duck genome. Distribution of ERV sequences on chromosomes in the Peking duck genome (A), Mallard genome (B) and Shaoxing duck genome (C). The intensity of the color represents the number of ERVs in the corresponding area.

Since the scale of chromosomes is very large, there is little difference in mapping by taking the entire length, midpoint, start, and end points for small segments, and to simplify the analysis, the start of the ERV is plotted on the chromosome. Analysis of the 3 duck genomes showed that the distribution of ERV on chromosomes was similar, mainly on chromosomes 1, 2, and 3 and the sex chromosome. However, the distribution of ERV on the sex chromosomes of the Pekin duck and Mallard was higher than that of Shaoxing duck, and the distribution of ERV on chromosome 39 was more abundant in Shaoxing duck than in the Mallard (Fig. 3).

### Classification of ERVs

**Fig. 4:**
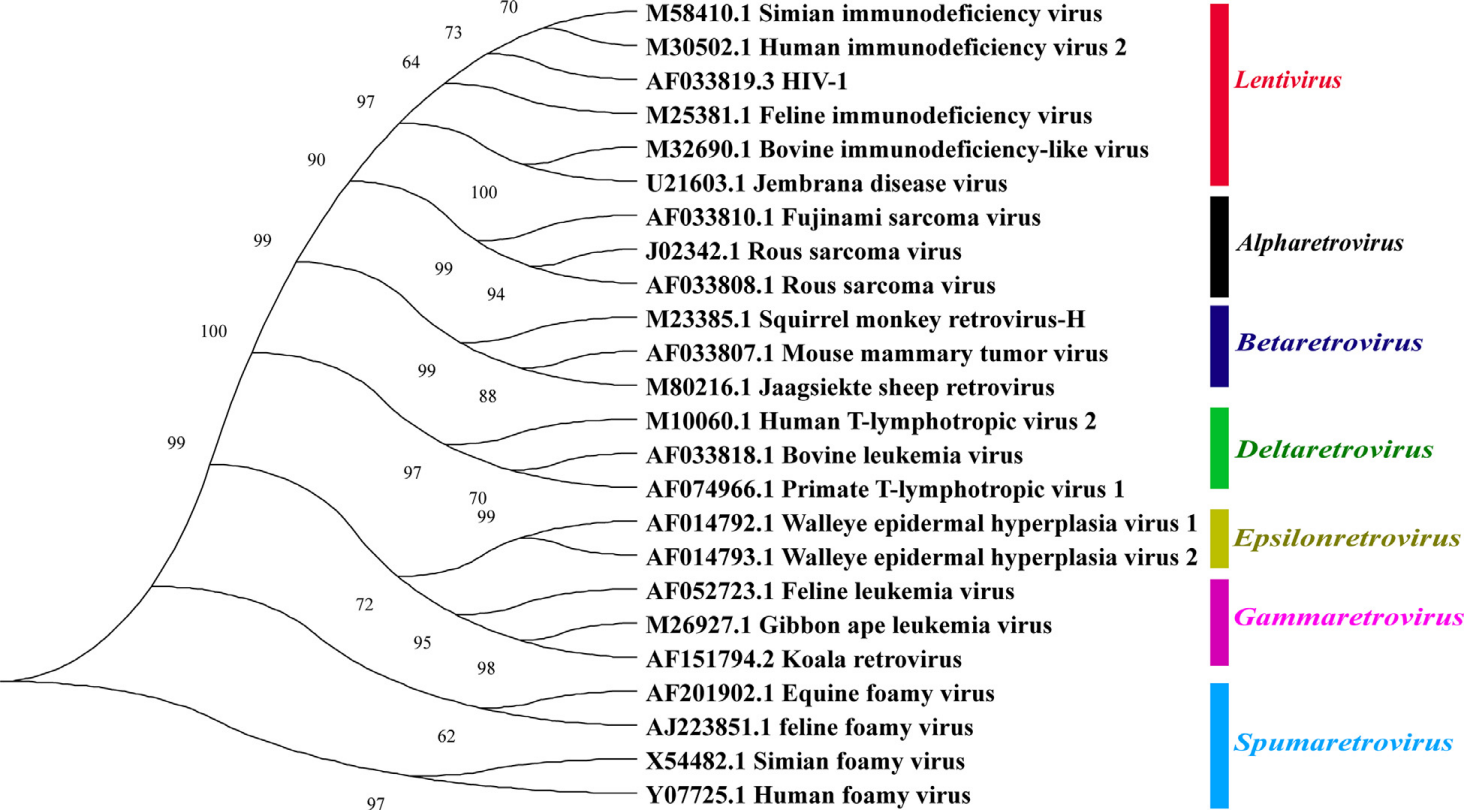
Phylogenetic relationships of different ERVs. The phylogenetic tree is based on the full-length sequences of 24 ERVs from seven genera.

**Fig. 5:**
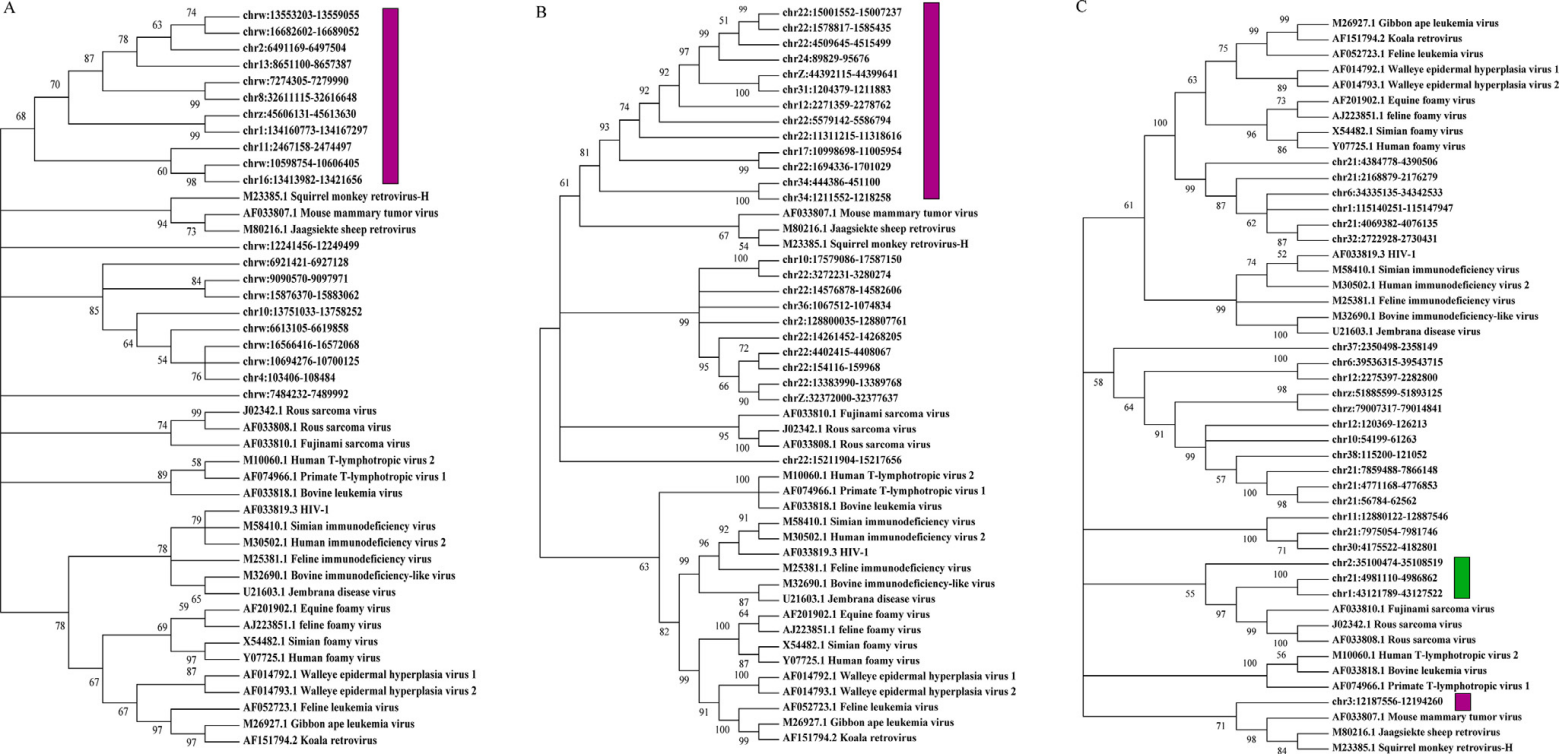
Classification of ERV sequences in the duck genome. Classification of ERV sequences in the Pekin duck genome (A), Mallard genome (B) and Shaoxing duck genome (C). Purple indicates *Alpharetrovirus* and green indicates *Betaretrovirus*.

Using the constructed evolutionary tree of 24 viral sequences with known complete structures, we found that retroviruses can be classified into seven genera, namely *Gamma-retrovirus, Epsilon-retrovirus, Spuma-retrovirus, Delta-retrovirus, Beta-retrovirus, Alpha-retrovirus* and *Lenti-virus* (Fig. 4). We constructed an evolutionary tree from selected ERV sequences and 24 reference virus sequences in the duck genome. The results showed that 11 ERV sequences were classified as *Betaretrovirus* (Fig. 5A) in the Peking duck genome, 13 ERV sequences were classified as *Betaretrovirus* (Fig. 5B) in the Mallard genome, 1 ERV sequence in the Shaoxing duck genome was classified as *Betaretrovirus* and 3 as *Alpharetrovirus* (Fig. 5C). By constructing a phylogenetic tree, we found more types of ERVs in Shaoxing duck, but their numbers were relatively small.

### Analysis of Potential DERVs Gene Integration Sites

**Fig. 6:**
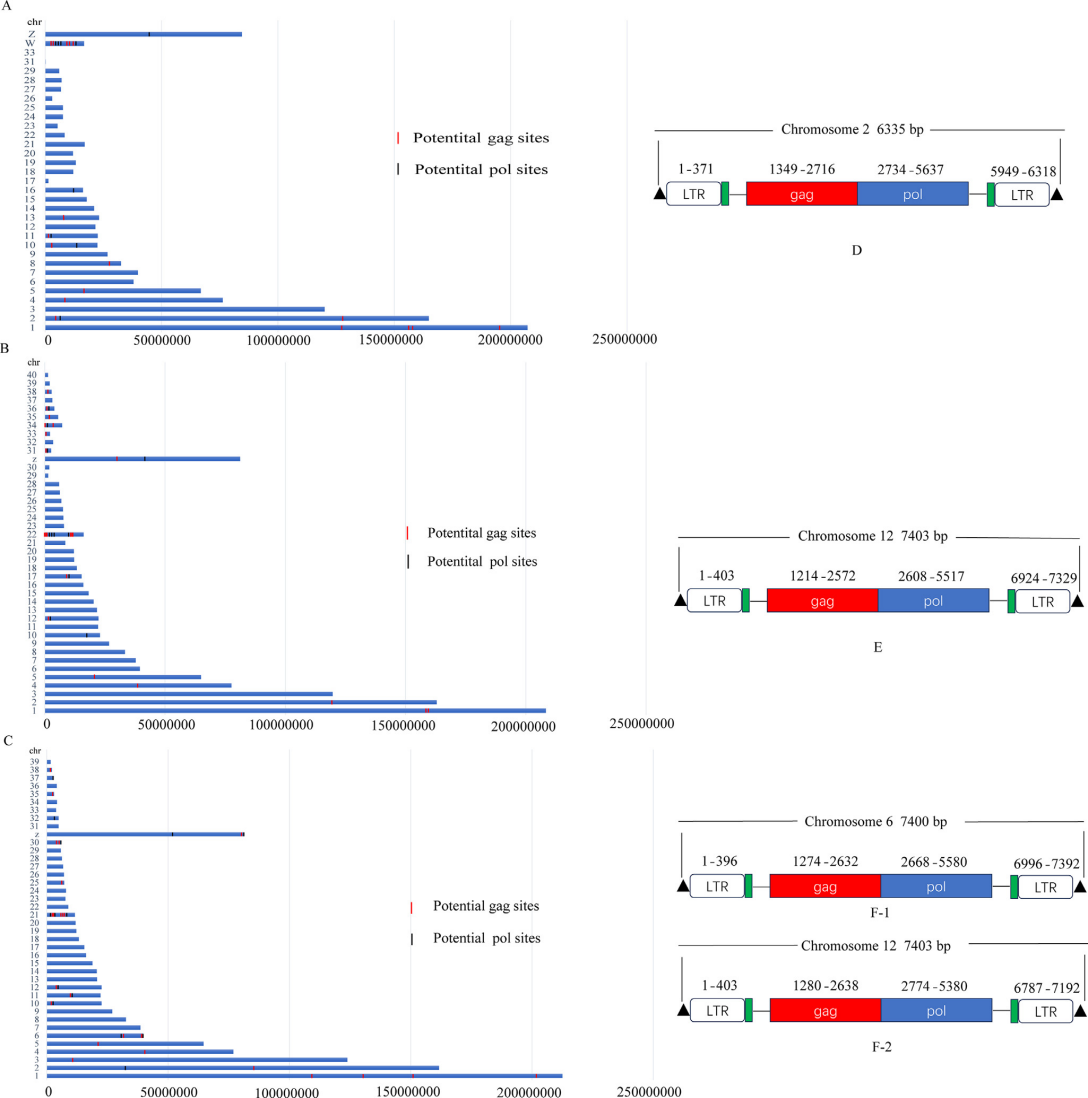
Chromosomal localization of the ERV element gene insertion site and structural demonstration of a relatively complete ERV. (A) Chromosomal localization of the insertion site of the ERV element gene in the Peking duck genome. (B) Chromosomal localization of the insertion site of the ERV element gene in the Mallard genome. (C) Chromosomal localization of the insertion site of the ERV element gene in the Shaoxing duck genome; Schematic representation of the relatively complete ERV structure in the Pekin duck genome (D), Mallard genome (E), and Shaoxing duck genome (F).

The Peking duck genome analysis showed 17 and 9 gag and pol genes, respectively. These genes were mainly distributed on chromosomes 1, 2, and W. The highest distribution on chromosome W was 9, including 5 gag genes and 4 pol genes (Fig. 6A), whereas in the analysis of the Mallard genome, it was found that there were 20, 12 gag and pol genes, respectively. These genes were mainly distributed on chromosomes 22 and 34. The highest distribution was found on chromosome 22 with 10, including 6 gag genes and 4 pol genes (Fig. 6B). The analysis of the Shaoxing duck genome showed 25, 13 gag and pol genes, respectively. These genes were mainly distributed on chromosomes 1, 6, and 21. The highest distribution was found on chromosome 21 with 8 genes, including 6 gag genes and 2 pol genes (Fig. 6C).

For completeness and accuracy, we screened endogenous retroviral sequences for relative structural integrity, that is, containing 2 major genes (gag and pol genes), 2 replication-critical sites (PBS and PPT), and the 5′LTR and 3′LTR. Surprisingly, we found only 1 endogenous retroviral sequence in the Pekin and Mallard genomes with a relatively structurally intact endogenous retroviral sequence. The position of this sequence in the Peking duck genome is chr2: 6,491,169 to 6,497,504 bp (Fig. 6D), and in the Mallard genome, it is chr12: 2,271,359 to 2,278,762 bp (Fig. 6E). In contrast, 2 endogenous retroviral sequences with relative structural integrity were found in the Shaoxing duck genome at chr6: 39,536,315 to 39,543,715 bp (Fig. 6 F1) and chr12: 2,275,397- 2,282,800 bp (Fig. 6 F2), respectively.

### Functional Enrichment of ERVs Neighboring Genes

**Fig. 7:**
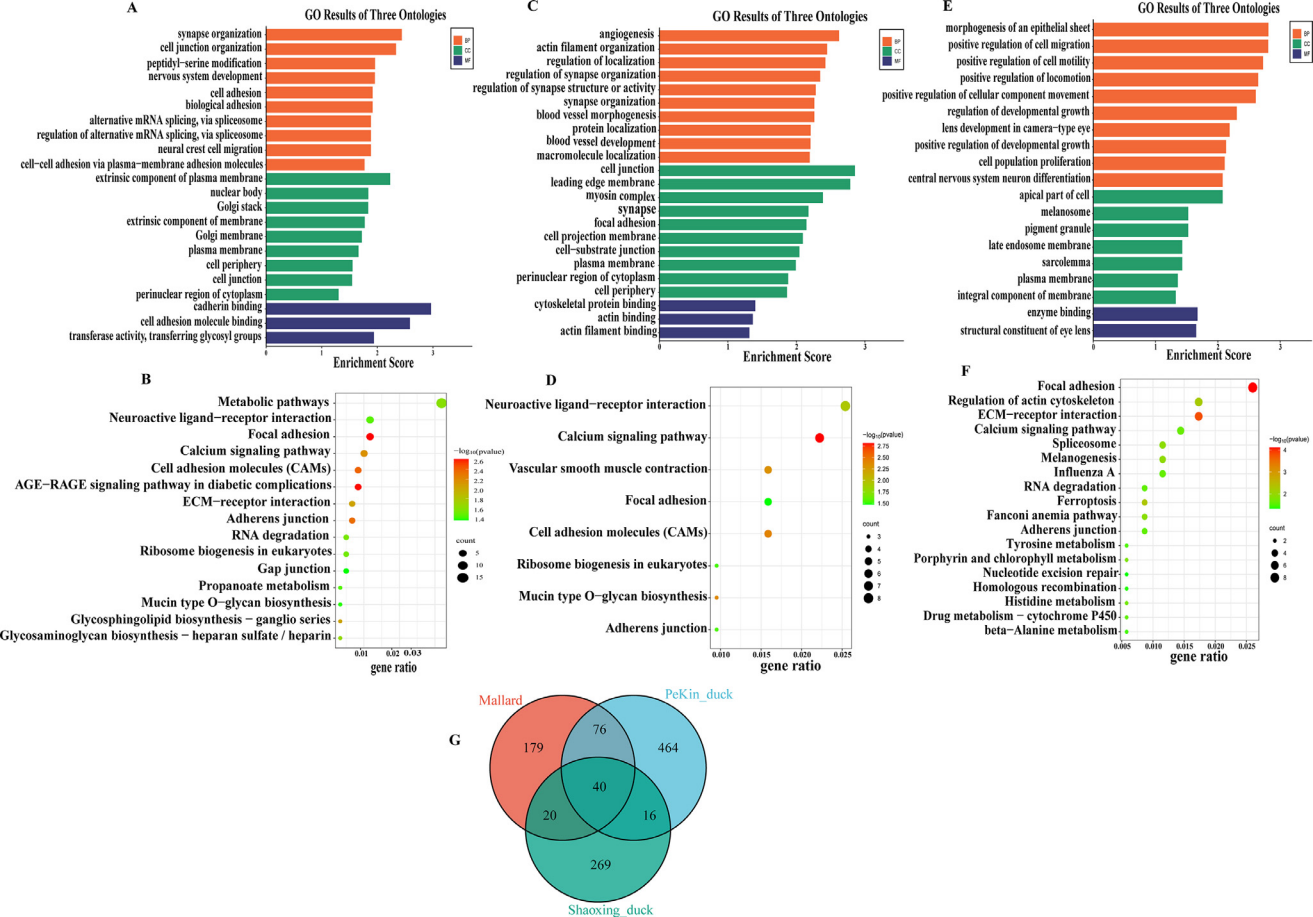
Enrichment analysis of ERV-adjacent genes in the duck genome. (A) Gene ontology (GO) annotation of ERV neighboring genes in the Peking duck genome. (B) Kyoto Encyclopedia of Genes and Genomes (KEGG) enrichment scatter plot of ERV neighboring genes in the Peking duck genome. (C) Gene ontology (GO) annotation of ERV neighboring genes in the Mallard genome. (D) KEGG enrichment scatter plot of ERV neighboring genes in the Mallard genome. (E) Gene ontology (GO) annotation of ERV neighboring genes in the Shaoxing duck genome. (F) KEGG enrichment scatter plot of ERV neighboring genes in the Shaoxing duck genome. (G) Number of ERV neighboring genes in 3 duck genomes.

We identified 596, 315, and 343 genes adjacent to ERVs in the Pekin duck, Mallard, and Shaoxing duck genomes, respectively (Table S5). Among these, 40 genes were common to all 3 duck genomes (Fig. 7G). To compare the functions of genes in the vicinity of different ERVs, we performed GO functional enrichment analysis on the genes found in the different duck genomes screened. Our experimental data were analyzed on the www.bioinformatics.com.cn. The results showed 21 top GO (*P <* 0.05) terms in the Pekin duck genome (Fig. 7A). Within the biological process category, “synapse organization” “cell junction organization” and “peptidyl-serine modification” were counted as the top 3 categories; In addition, in the cellular component category, genes were enriched in the following terms “extrinsic component of plasma membrane” “cell junction” and “Golgi stack.” In the molecular function category, “cadherin binding” “cell adhesion molecule binding” “transferase activity, transferring glycosyl groups” terms were significant.

There are 23 GO terms (*P <* 0.05) in the Mallard genome (Fig. 7C). The results of GO showed that most of the genes were enriched in the following biological process terms, namely, “angiogenesis” “actin filament organization” and “regulation of localization” was significantly higher; In addition, “cell junction” “leading edge membrane” and “myosin complex” were enriched in the cellular component terms; in the molecular function, “cytoskeletal protein binding” “actin binding” and “actin filament binding” were enriched.

There are 19 significant GO terms (*P <* 0.05) in the Shaoxing duck genome (Fig. 7E). Within the biological process category, “morphogenesis of an epithelial sheet” “positive regulation of cell migration” and “positive regulation of locomotion” were enriched; In addition, “apical part of cell” “melanosome” “pigment granule” were enriched in the cellular component; whereas “enzyme binding” and “structural constituent of eye lens” were enriched in the molecular function term.

### KEGG Pathway Analysis of ERV-Adjacent Genes

Based on the KEGG enrichment analysis of the neighboring genes of ERV in the Pekin duck genome, the top 14 significant signaling pathways (*P <* 0.05) are shown in (Fig. 7B). These pathways included Metabolic pathways, AGE-RAGE signaling pathway in diabetic complications, and Cell adhesion molecules (**CAM**). Similarly, the top 8 significantly enriched signaling pathways (*P <* 0.05) of the ERV-adjacent genes in the Mallard genome are shown in (Fig. 7D). These pathways included the Calcium signaling pathway, Vascular smooth muscle contraction, Cell adhesion molecules, etc. Moreover, the KEGG enrichment of the top18 significantly enriched signaling pathways (*P <* 0.05) of the ERV-adjacent genes in the Shaoxing duck genome are shown in (Fig. 7F). These pathways included Focal adhesion, Regulation of actin cytoskeleton, ECM-receptor interaction, etc.

### Expression of Overlapped Genes in Tissues

**Fig. 8:**
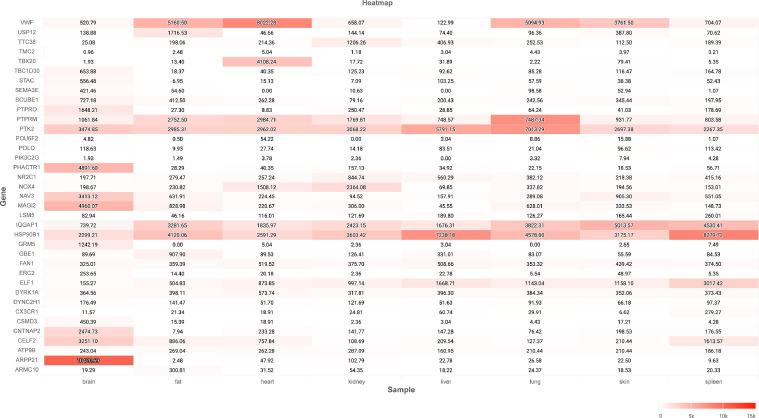
Expression status of shared ERVs nearby genes in 8 tissues of 8-wk-old Mallard.

According to the gene expression profiling results, the overlapped genes (Fig. 7G) were highly expressed in 8 tissues of 8-wk-old Mallard, including brain, fat, heart, kidney, liver, lung, skin, and spleen. Notably, *ARPP21* was the most highly expressed gene in the brain, followed by *VWF* in fat, and *PTPRM* in the lung tissues (Fig. 8). The study revealed the significant expression of these genes in the various tissues and proposed a new approach to analyze the function of ERV.

## DISCUSSION

ERVs are inserted into animal genomes, and over millions of years of evolution and evolution, a portion of the sequence is lost, they are powerful agents of genome evolution and phenotypic diversity, and they can give rise to a diversity of genetic variation. In this study, we explored the essential features of ERVs in 3 different duck reference genomes, including comparisons of number, structure, distribution, and class. Specifically, 1,607, 2,031, and 1,908 ERVs were retrieved in the Pekin duck, Mallard and Shaoxing duck genomes. Domestic duck breeds arise from the mallards (Guo et al., 2021). ERVs were integrated earlier in Mallard, so the relatively small number of identified ERVs in the genomes of Pekin duck and Shaoxing duck aligns with logical reasoning. The number of ERVs with significant integrity throughout the entire duck genome is low. Only one relatively complete ERV structure was identified in the genomes of both the Pekin duck and Mallard, while 2 relatively complete ERV structures were identified in the genome of the Shaoxing duck. However, Dai et al., (2022) detected over 400 ERVs with relatively intact coding sequences in the chicken genome, employing the same identification method. The length of DERVs is primarily focused within the 4,000 to 8,000 bp range, which is generally consistent with the classical length of ERVs. However, there is still a large proportion of sequences with lengths less than 4000 bp. According to the hypothesis posited by some scholars (Wang et al., 2019), ERV sequences undergo inactivation due to the accumulation of nonsense mutations, insertions, and deletions in both their internal coding regions and long terminal repeats over time. This results in a variety of truncation patterns. Endogenous retroviral sequences exhibited the most substantial internal gene deletions. Our findings suggest that the distribution of DERVs among chromosomes is not uniform and may be linked to the random nature of ERV insertions. It also gives it the potential to influence all aspects of the host's physiological activity (Bolisetty et al., 2012). But this contrasts a previous study by Wragg et al., (2015), which indicated that EAV-HP is nonrandomly distributed throughout the genome and may play a vital role in species adaptation. *Betaretrovirus* was detected in 3 duck genomes, with a comparatively lower quantity found in the Shaoxing duck genome. Notably, the Shaoxing duck genome demonstrated the presence of *Alpharetrovirus*. This observation triggers an explanation for this phenomenon. One possible reason is that it is a fortuitous situation for the small number of ERVs selected in the 3 duck genomes. Alternatively, it could mean that *Alpharetrovirus* has a special evolutionary significance in the genome of the Shaoxing duck, possibly involving the influence of different retrovirus families on genome stability.

Research on the potential function of ERVs. In the early stages, some scholars discovered that a 7.5kb ALV reverse insertion within intron 4 of the *TYR* gene caused recessive white plumage in chickens (Chang et al., 2007); the integration of a 4.2kb EAV-HP in the 5′UTR region of the *SLCO1B3* gene on chicken chromosome 1 resulted in green-shell egg phenotype (Wang et al., 2013); Li et al., (2019a) found that the integration of ERV in the 5′UTR region of *CYP19A1* gene on chicken chromosome 10 led to the henny feather characteristics. We discovered several trait-related genes in the endogenous retroviral neighboring genes of Pekin duck. *PAN3* and *QKI* are candidate genes for the greenhead trait in Nonghua hemp-plumaged male ducks, a protein-interaction effect exists between these genes and pigmented coloring genes (Wang et al., 2023). Furthermore, The *BLVRA* gene is associated with the major pigmentation of eggshells (Chen et al., 2016). The *PTPRT* gene affects the growth rate of primary wing feathers (Ma et al., 2023), while the *IMMP2L* gene is a significant factor in influencing through-tube traits of duck feathers (Ma et al., 2023). Some nearby genes of endogenous retroviruses that can affect traits have been identified in the in Mallard. For instance, the *GBIE* gene is associated with duck weight at 42 d (Guo et al., 2022). *ABCG2* gene affects eggshell color due to its membrane transporter function for biliverdin (Liu, et al., 2021). Genes, including *PAN3* and *BLVRA*, which impact the organism's traits, were detected in the neighboring genes of ERVs in the Shaoxing duck. The insertion of ERVs may affect the expression of these genes, but experiments are needed to verify this further. In general, ERVs have diverse functions and play important roles in biological evolution and gene regulation.

## CONCLUSIONS

We characterized the general features of ERVs in 3 different duck genomes. The results showed that ERVs were most abundant in the Mallard genome and showed a nonrandom distribution with a severe loss of structural integrity in the duck genome. In addition, ERV neighboring genes were enriched to a high number of GO, suggesting that ERV may play a broad role in regulating gene function. Our study reveals the general characterization of DERVs in the genome and their potential biological functions, which provides valuable information for future exploration in this field.

## Appendix Supplementary materials

Image, application 1

## Footnotes

Supplementary material associated with this article can be found in the online version at doi:10.1016/j.psj.2024.103543.

## References

[bib0001] Bannert N., Kurth R. (2006). The evolutionary dynamics of human endogenous retroviral families. Annu. Rev. Genomics Hum. Genet..

[bib0002] Bolisetty M., Blomberg J., Benachenhou F., Sperber G., Beemon K. (2012). Unexpected diversity and expression of avian endogenous retroviruses. MBio.

[bib0003] Chang C., Furet J., Coville J., Coquerelle G., Gourichon D., Tixier-Boichard M. (2007). Quantitative effects of an intronic retroviral insertion on the transcription of the tyrosinase gene in recessive white chickens. Anim. Genet..

[bib0004] Chen L., Huang X., Tian Y., Tao Z., Lu L. (2016). Identifying genes associated with blue eggshell in ducks (Anas platyrhynchos domesticus) by transcriptome analysis. Chin. J. Agric. Biotechnol. Chinese..

[bib0005] Cui J., Zhao W., Huang Z., Jarvis E.D., Gilbert M.T.P., Walker P.J., Holmes E.C., Zhang G. (2014). Low frequency of paleoviral infiltration across the avian phylogeny. Genome Biol..

[bib0006] Dai M., Xie T., Feng M., Zhang X. (2022). Endogenous retroviruses transcriptomes in response to four avian pathogenic microorganisms infection in chicken. Genomics..

[bib0007] Ellinghaus D., Kurtz S., Willhoeft U. (2008). LTRharvest, an efficient and flexible software for de novo detection of LTR retrotransposons. BMC Bioinf..

[bib0008] Galindo-González L., Mhiri C., Deyholos M.K., Grandbastien M.-A. (2017). LTR-retrotransposons in plants: engines of evolution. Gene..

[bib0010] Guo Q., Huang L., Jiang Y., Wang Z., Bi Y., Chen G., Bai H., Chang G. (2022). Genome-wide association study of feed efficiency related traits in ducks. Animals..

[bib0009] Guo X., He X.-X., Chen H., Wang Z.-C., Li H.-F., Wang J.-X., Wang M.-S., Jiang R.-S. (2021). Revisiting the evolutionary history of domestic and wild ducks based on genomic analyses. Zool. Res..

[bib0011] Jern P., Coffin J.M. (2008). Host-retrovirus arms race: trimming the budget. Cell Host Microbe..

[bib0012] Johnson W.E. (2019). Origins and evolutionary consequences of ancient endogenous retroviruses. Nat. Rev. Microbiol..

[bib0013] Kumar S., Stecher G., Tamura K. (2016). MEGA7: molecular evolutionary genetics analysis version 7.0 for bigger datasets. Mol. Biol. Evol..

[bib0014] Li J., Davis B.W., Jern P., Dorshorst B., Siegel P.B., Andersson L. (2019). Characterization of the endogenous retrovirus insertion in CYP19A1 associated with henny feathering in chicken. Mobile DNA..

[bib0015] Li S., Hu X., Tian R., Guo Y., Chen J., Li Z., Zhao X., Kuang L., Ran D., Zhao H. (2019). RNA-Seq-based transcriptomic profiling of primary interstitial cells of Cajal in response to bovine viral diarrhea virus infection. Vet. Res. Commun..

[bib0016] Liu H., Hu J., Guo Z., Fan W., Xu Y., Liang S., Liu D., Zhang Y., Xie M., Tang J. (2021). A single nucleotide polymorphism variant located in the cis-regulatory region of the ABCG2 gene is associated with mallard egg colour. Mol. Ecol..

[bib0017] Ma S., Li P., Liu H., Xi Y., Xu Q., Qi J., Wang J., Li L., Wang J., Hu J. (2023). Genome-wide association analysis of the primary feather growth traits of duck: identification of potential Loci for growth regulation. Poult. Sci..

[bib0018] Menéndez-Arias L., Sebastián-Martín A., Álvarez M. (2017). Viral reverse transcriptases. Virus Res..

[bib0019] Mourikis T.P., Aswad A., Katzourakis A., Kliman R.M. (2016). Encyclopedia of Evolutionary Biology.

[bib0020] Patil S., Khune V., Karunamay S. (2020). Duck farming: A potential to reduce poverty in rural households in Indian communities–a review. Pharm. Innov. J..

[bib0021] Steinbiss S., Willhoeft U., Gremme G., Kurtz S. (2009). Fine-grained annotation and classification of de novo predicted LTR retrotransposons. Nucleic Acids Res..

[bib0022] Wang J., Jiang S., Xi Y., Qi J., Ma S., Li L., Wang J., Bai L., He H., Xu H. (2023). Integration of GWAS and eGWAS to screen candidate genes underlying green head traits in male ducks. Anim. Genet..

[bib0023] Wang Z., Qu L., Yao J., Yang X., Li G., Zhang Y., Li J., Wang X., Bai J., Xu G. (2013). An EAV-HP insertion in 5′ flanking region of SLCO1B3 causes blue eggshell in the chicken. PLos Genet..

[bib0024] Wang X., Wang B., Liu Z., Garber P.A., Pan H. (2019). Genome-wide characterization of endogenous retroviruses in snub-nosed monkeys. PeerJ..

[bib0025] Wragg D., Mason A.S., Yu L., Kuo R., Lawal R.A., Desta T.T., Mwacharo J.M., Cho C.-Y., Kemp S., Burt D.W. (2015). Genome-wide analysis reveals the extent of EAV-HP integration in domestic chicken. BMC Genom..

[bib0026] Zhu F., Yin Z.-T., Wang Z., Smith J., Zhang F., Martin F., Ogeh D., Hincke M., Lin F.-B., Burt D.W. (2021). Three chromosome-level duck genome assemblies provide insights into genomic variation during domestication. Nat. Commun..

